# The Effect of Polyhydramnios on Cervical Length in Twins: A Controlled Intervention Study in Complicated Monochorionic Pregnancies

**DOI:** 10.1371/journal.pone.0003834

**Published:** 2008-12-02

**Authors:** Neelam Engineer, Keelin O'Donoghue, Ruwan C. Wimalasundera, Nicholas M. Fisk

**Affiliations:** 1 Centre for Fetal Care, Queen Charlotte's and Chelsea Hospital, London, United Kingdom; 2 Institute of Reproductive and Developmental Biology, Division of Surgery, Oncology, Reproduction and Anaesthesia, Faculty of Medicine, Imperial College London, London, United Kingdom; 3 University of Queensland Centre for Clinical Research, Brisbane, Queensland, Australia; Institute for Clinical Effectiveness and Health Policy (IECS), Argentina

## Abstract

**Objective:**

To test the hypothesis that cervical shortening in polyhydramnios reflects the degree of excess amniotic fluid, and increases with normalisation of amniotic fluid volume.

**Study Design:**

Prospective cohort study of 40 women with monochorionic twins undergoing interventional procedures between 16–26 weeks. Cervical length was assessed via transvaginal sonography pre-procedure, 1 and 24 hours post-procedure, and results compared between amnioreduction and control procedures. Amniotic fluid index (AFI) was measured pre- and post-procedure.

**Results:**

Pre-procedural cervical length correlated with AFI (linear fit = 5.07 -0.04x, R^2^ = 0.17, *P* = 0.03) in patients with polyhydramnios (n = 28). Drainage of 2000ml fluid (range 700–3500ml), reduced AFI from 42cm to 21cm (*P*<0.001). Their pre-procedural cervical length did not change at one (mean Δ:−0.1cm, 95%CI, −0.4 to 0.2) or 24 hours (0.2cm, −0.1 to 0.6) after amnioreduction. There was no change in cervical length at control procedures.

**Conclusion:**

Cervical shortening in twins with polyhydramnios does not appear to be an acute process; cervical length can be measured before or after therapeutic procedures.

## Introduction

Cervical length has been extensively studied as a predictive tool to identify women at high risk of preterm delivery in both singleton and multiple pregnancies. The inverse relationship between cervical length and preterm delivery is well documented [1]; [2]. Uncontrolled studies first suggested that cervical length in women with multiple pregnancy was shorter than singleton pregnancies [3]; [4]. A small controlled study showed that twin cervices were 5 mm shorter and triplets 10mm shorter at 23–24 weeks, this disparity increasing with gestational age [5]. Nevertheless, when predicting preterm delivery in twin pregnancies, a higher cervical length cut-off than in singletons has been recommended because of the greater prevalence of preterm delivery. Thus, a cut-off of 25mm achieves a sensitivity of 80% to predict preterm delivery ≤30 weeks gestation, which equates to the sensitivity seen with a cervical length ≤15mm in singletons [6]. Although the mechanism of preterm labour and premature cervical maturation in multiples is not well understood, it is widely attributed to increased uterine volume and myometrial stretch leading to premature cervical ripening and ultimately preterm delivery.

Uterine overdistension can also occur in singleton pregnancies with polyhydramnios. It is known that amniotic pressure rises when the amniotic fluid index (AFI) exceeds 40 [7], but the only study of singletons with polyhydramnios (albeit largely mild as the mean AFI was only 28cm) failed to find any inverse correlation between cervical length and AFI [8]. The effect of AFI on cervical length in twin pregnancies however has not been studied.

Monochorionic multiple pregnancies complicated by twin to twin transfusion syndrome (TTTS ) are at high risk of preterm delivery, due both to the effect of raised amniotic pressure with two or more fetuses and excessive amniotic fluid volume [7], and to the iatrogenic effects of therapeutic procedures. Cervical length prior to fetoscopic laser ablation has been shown to predict miscarriage and preterm labour, with values <30mm having odds ratios of 2.2–3.5 for delivery before 28 and 34 weeks respectively [9]. The main positive predictive value was seen in the 7% with values <20mm. However, it is unclear whether cervical shortening under these circumstances is simply an acute effect of uterine overdistension, or a more chronic process reflecting longstanding uterocervical pathology.

We hypothesized (i) that cervical shortening in complicated monochorionic twin pregnancies reflects the degree of polyhydramnios, and (ii) that normalization of amniotic fluid volume in patients with polyhydramnios leads to lengthening of the cervix. We tested this in a prospective cohort study using the controlled intervention model afforded by twin pregnancies undergoing invasive procedures, comparing those with or without amnioreduction.

## Results

### 1. Clinical details

Forty four women were recruited, of whom 4 did not complete the study. Clinical characteristics of the study population are shown in Table 1. Only one woman had a previous history of preterm delivery before 34 weeks. The indication for intervention was TTTS in the majority of cases (90%) and discordant fetal anomaly in the remainder. Of 40 pregnancies with complete data, 36 had TTTS (6 stage I, 4 stage II, 23 stage III 3 stage IV) and 4 discordant fetal anomaly. One patient with discordant fetal anomaly also had stage III TTTS with polyhydramnios and underwent bipolar cord occlusion with concomitant amnioreduction. Overall, 29 women underwent selective fetoscopic laser ablation (20 with and 9 without concomitant amnioreduction), 7 primary amnioreduction and 4 selective feticide by bipolar cord occlusion (1 with and 3 without concomitant amnioreduction).

**Table 1:** Maternal characteristics and indications for procedure

Parameter	Group A	Group B	Number	*P*
Number recruited	12	28	40	
Maternal age in yrs^a^	25 (21–40)	34 (22–42)		ns
Gestational age at procedure in weeks^a^	19 (16–26)	21 (17–26)		ns
**Parity** b				
Nulliparous	7 (58)	13 (46)	20 (50)	ns
Primiparous	3 (25)	11 (39)	14 (35)	ns
Multiparous	2 (17)	4 (14)	6 (15)	ns
**Indication for intervention** b				
1. TTTS	9 (75)	27 (96)	36 (90)	ns
2. Discordant anomaly	3 (25)	1 (4)	4 (10)	ns
**Type of intervention** b				
1. Amnioreduction		7 (25)	7 (17)	
2. Laser ablation	9 (75)	20 (71)∗	29 (73)	ns
3. Bipolar cord occlusion	3 (25)	1 (3)∗ #	4 (10)	ns
**Amniotic fluid index** c				
Pre-procedure	23±5.8	42±10.2		0.0001
Post procedure	20±4.9	21±5.8		ns
**Maximum vertical pocket in recipient sac** c				
Pre-procedure	7.1±2.0	11.9±2.8		0.0001
Post-procedure	6.1±1.6	6.5±1.2		ns

a Data shown as median (range)

b Data shown as N (%)

c Data shown as mean±SD

∗ with concomitant amnioreduction

# pregnancy complicated by both discordant anomaly and stage III TTTS

Patients were classified into two groups depending upon whether excess amniotic fluid was removed at the time of procedure. Group A (control group) comprised women undergoing control procedures, which did not involve removal (or infusion) of amniotic fluid. Group B (study group) comprised women who had amnioreduction performed at the time of procedure, implying a reduction in uterine distension. Table 1 details the indications and interventions in each group.

### 2. Correlation with AFI and cervical length

**Figure 1:**
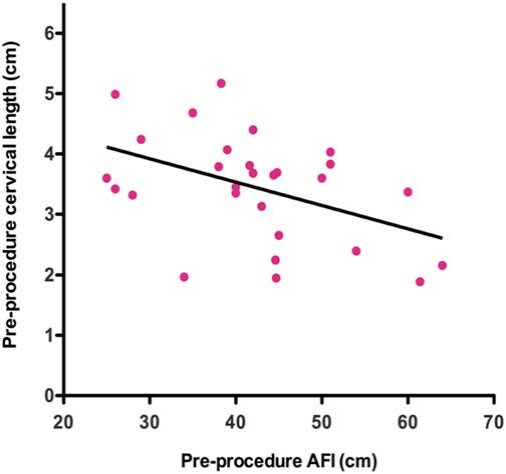
Inverse linear relationship between pre-procedural cervical length (y) and pre-procedural AFI (x) in group B. y = 5.07 -0.04x, R^2^ = 0.17, *P* = 0.03.

AFI increased with gestational age within the window studied (r = 0.56, *P* = 0.0002). In group A, we found no relationship between pre-procedure cervical length and AFI. However, in group B, cervical length decreased significantly with increasing AFI (Linear fit = 5.07 -0.04x, R^2^ = 0.17, *P* = 0.03) (Figure 1). Although the variance was considerably greater in those with polyhydramnios, cervical length was nevertheless similar in the two groups (3.5±0.4cm and 3.4±0.9),

### 3. Change in AFI and cervical length over time

In group A, the AFI did not significantly change after the procedure (mean Δ: −3.3cm, 95% CI, −1.4 to −4.3. Pre-procedural cervical length did not change either at one hour (mean Δ: −0.3cm, 95% CI, −0.5 to 0.1) or 24 hours (0.2cm,−0.2 to 0.5) (Table 2).

**Table 2:** Cervical length measurements with mean Δ 1 hour and 24 hour post procedure

Cervical length	Group A Mean±SD (cm)	Mean Δ, 95% CI	Group B Mean±SD (cm)	Mean Δ, 95% CI
Pre-procedure	3.5±0.4		3.4±0.9	
1 hour post procedure	3.3±0.4	−0.3, −0.5 to 0.1	3.4±0.8	−0.1, −0.4 to 0.2
24 hour post procedure	3.7±0.7	0.2, −0.2 to 0.5	3.8±0.8	0.2, −0.1 to 0.6

|n group B, amnioreduction of 2000ml (median, range 700–3500ml) resulted in mean AFI falling from 42 to 21cm (mean Δ −20.9cm, 95% CI, −17.6 to −24.1, *P*<0.0001). However, this reduction in uterine volume did not increase the pre-procedural cervical length at 1 hour (mean Δ: −0.1cm, 95% CI, −0.4 to 0.2) or 24 hours (0.2cm, −0.1 to 0.6) (Table 2). Further, the change in cervical length at 1 and 24 hours did not differ significantly between control and study groups (Figure 2). Overall, we found no significant association between pre-procedure cervical length and parity, underlying disease or type of interventional procedure.

**Figure 2:**
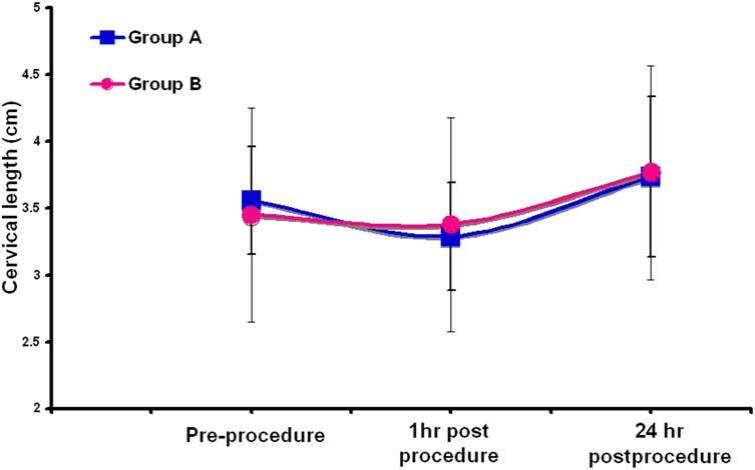
Mean cervical length was unchanged in the two groups over three time periods: pre-procedure, 1 hour post procedure and 24 hour post procedure. *Error* bars represent standard deviation.

## Discussion

This prospective cohort study evaluates the relationship between amniotic fluid volume and cervical length in twin pregnancies with polyhydramnios. We report an inverse relationship between cervical length and AFI (Linear fit = 5.07 -0.04x, *P* = 0.03) in patients with polyhydramnios necessitating amnioreduction. This finding supports our first hypothesis but contrasts with data from singleton pregnancies by Hershkowitz et al where cervical length did not decline with increasing amniotic fluid volume [8]. This difference may be attributed to a greater degree of polyhydramnios and uterine overdistension in our study group (mean AFI = 42cm) compared to Hershkowitz et al's cohort of singleton pregnancies where the mean AFI was only 28cm [8]. The lack of association between the cervical length and AFI in our control group was expected, as the mean AFI was only 23cm.

We employed AFI as a semi-quantitative method of amniotic fluid volume estimation as it is the most widely accepted parameter for quantifying the degree of polyhydramnios in both twins and singletons [10] and a better measure of uterine overdistension than DVP in twins which only quantifies the amniotic fluid in an individual sac. Global AFI in diamniotic twin pregnancies increases linearly with gestational age [11] while in amnioreduction experiments our group has reported a linear relationship between AFI reduction and volume drained in both twin and singleton pregnancies [12].

Since there was a significant association between pre-procedure cervical length and AFI, and since AFI fell markedly with amnioreduction as anticipated (mean Δ = 20cm), we expected the cervix to lengthen post-procedure. However, this was not demonstrated in our study; cervical length did not change post-procedure either acutely or subacutely at 24 hours. Possible explanations are that cervical shortening in the presence of polyhydramnios is a chronic irreversible process, possibly reflecting the duration of polyhydramnios, or maybe other factors such as the underlying disease.

It is difficult to speculate from our study period of only a day whether cervical lengthening may have occurred at a later time period, ie. a week or weeks after amnioreduction. Our 1 hour and 24 hour post procedure measurements were timed to coincide with routine post intervention clinical evaluations. Empirically, delayed measurements were available 3–7 days post procedure in 3 patients, with a minimal median change of −0.1cm. Another potential explanation for the lack of change with reduction in amniotic fluid volume is that we had few patients with short cervices (only 21% had lengths <2.5cm). Notwithstanding this, we nevertheless demonstrated an association between cervical length and AFI in those with excessive amniotic fluid volume, in whom AFI accounted for 17% of the variance in cervical length. Indeed, our mean +/− SD cervical length measurement in Group B was almost identical to that reported in the study of TTTS patients prior to treatment in which cervical length was shown to be the main predictor of very preterm delivery [13]. Finally, Type II error is an unlikely explanation for our negative findings as post hoc analysis using the pre-amnioreduction SD in group B indicates that n = 28 would detect greater than a 0.4cm change on one tailed t-testing with α of 0.05 and 1-β of 0.8.

We conclude that there was no acute effect of amnioreduction on cervical length and speculate that polyhydramnios *per se* is not a simple physical mediator of decreased cervical length. This negative finding has practical clinical implications for singleton and twin pregnancies undergoing amnioreduction to reduce uterine overdistension and subsequent risk of preterm labour and delivery. Amnioreduction, while relieving maternal discomfort, does not necessarily reduce preterm delivery rates. The Eurofetus trial showed that amnioreduction for treatment of TTTS not only confers no benefit but is associated with a higher preterm delivery rate compared to fetoscopic laser ablation (69% versus 42% for delivery before 32 weeks gestation respectively) [14]. Future studies of changes in cervical length in response to amnioreduction in singletons may prove difficult, due to the rarity of singleton polyhydramnios of sufficient severity to warrant amnioreduction [15].

In terms of more chronic exposure to increased amniotic fluid volume, it is not known whether medical amnioreduction using sulindac or indomethacin increases cervical length. Although sulindac reduces mean AFI by 40%, [16] it does not reduce preterm labour [17]. Indomethacin has been associated in an individual patient meta-analysis with an 86% decrease in preterm births before 24 weeks gestation when used in asymptomatic women with short cervix less than 25mm [18]. However, any change on cervical length due to use of these non selective prostaglandin H synthase inhibitors may solely be attributed to their tocolytic effect rather than reduced amniotic volume.

Robyr *et al* reported that short cervical length before treatment was an independent risk factor for preterm delivery in TTTS treated by fetoscopic laser. In that study, cervical length <30mm was associated with a preterm delivery rate of 74% before 34 weeks gestation [9]. Although further studies are required to establish the role of cervical length screening in TTTS, the main clinical implication of our results is that for prediction of preterm delivery it does not matter when the cervical length measurement is done; it may be performed either before or after an intervention.

### Conclusion

In complicated monochorionic twin pregnancies, shorter cervical length correlated with the degree of polyhydramnios but cervical length did not lengthen within 24 hours of restoration of normal amniotic fluid volume. We conclude that cervical shortening in the presence of polyhydramnios does not appear to be an acute process and thus clinically the cervix can be measured either before or after therapeutic procedures which normalize amniotic fluid volume.

## Methods

We enrolled women below 26 weeks gestation with monochorionic twin pregnancies undergoing clinically indicated invasive procedures at the Centre for Fetal Care, Queen Charlotte's & Chelsea Hospital. Procedures were indicated for treatment of TTTS or discordant fetal anomaly. Ethical approval was obtained from the institutional research ethics committee (REC number: 05/Q0406/90), and informed consent was obtained from each participant to participation in the research study after first securing written consent to the clinical procedure.

AFI was measured before and 1 hour post procedure by the standard technique summating the deepest pool in each of four quadrants [19]. As per convention in twins [11], we measured global AFI. The AFI was described in singletons as a technique to measure the total amniotic fluid volume within the uterus [19], and we applied the same technique to a uterus with twin pregnancy. Polyhydramnios was defined as AFI ≥25 cm [20]. In addition, the maximum vertical pocket (MVP) was measured in each sac to determine discordance in amniotic fluid volume. Cervical length was measured by transvaginal sonography by two investigators (NE, KOD). All patients were asked to empty their bladder before the examination, the vaginal probe (5–9MHz, Accuson Sequoia) was placed in the anterior fornix of the vagina [13];[21]–[24] and a sagittal view of the cervix with the echolucent endocervical mucosa demonstrated. Care was taken not to distort the cervix from undue pressure from the transducer. Longitudinal measurements of cervical length were obtained over three time periods: pre-procedure (within 24 hours prior to procedure), 1 hour post procedure and 24 hours later. At each time-period, images of 3 measurements were recorded over 3–5 minutes by one of two investigators (NE, KOD), and the mean calculated.

All images of cervical length were stored and reviewed in a subsequent blinded analysis to ensure reproducibility. Mean measurements of 20 randomly selected clips compared between the two investigators revealed 95% limits of agreement of −0.4 to 0.5 cm (SD of bias: 0.2, Bland Altman comparison) [25].

Gestational age was based on first trimester crown-rump length measurement in line with national policy in the UK. Interventional procedures were performed percutaneously under either local or regional anesthesia. TTTS was classified according to Quintero's staging system [26]. Treatment for TTTS included either amnioreduction for early stage disease (stage I and some stage II) or selective fetoscopic laser ablation of anastomotic vessels for more advanced disease (stage II/III/IV) and in the latter, excess amniotic fluid was drained at the end of the procedure using a 50ml syringe and measured in a sterile jug before discarding. Amnioreduction was indicated on clinical grounds when the AFI exceeded 40cm [10] or there was significant maternal discomfort secondary to uterine overdistension in cases with an AFI between 30–40cm. In cases of discordant fetal anomaly with normal amniotic fluid volume, selective feticide of the affected fetus was performed on parental request using bipolar cord occlusion without concomitant amnioreduction. All women were admitted overnight for observation and repeat cervical length measurements were performed the following day.

### Statistical analysis

Statistical analysis was performed using Prism version 5.0 (Graphpad, San Diego, USA) and Analyse it® (Leeds, UK) software. Normal distribution of data was established using D'Agostino-Pearson normality test. Delta (Δ) values were defined as post-procedural minus pre-procedural values. Paired t test was used for parametric data and Fisher exact test to analyze categorical data. One way and two way ANOVA were performed to compare variables between the two groups. Linear regression was done by the least squares method, and Pearson's correlation co-efficient determined. *P* value <0.05 was considered significant.
